# Mechanism of *Abelmoschus manihot* L. in the Treatment of Contrast-Induced Nephropathy on the Basis of Network Pharmacology Analysis

**DOI:** 10.3389/fneph.2022.834513

**Published:** 2022-04-22

**Authors:** Zhongchi Xu, Lichao Qian, Ruge Niu, Yibei Wang, Ying Yang, Chunling Liu, Xin Lin

**Affiliations:** ^1^ Jiangsu Province Hospital of Chinese Medicine, Affiliated Hospital of Nanjing University of Chinese Medicine, Nanjing, China; ^2^ Nanjing Hospital of Chinese Medicine, Affiliated Hospital of Nanjing University of Chinese Medicine, Nanjing, China

**Keywords:** contrast-induced nephropathy, Abelmoschus manihot L., network pharmacology, molecular docking, total flavonoids

## Abstract

**Background:**

Contrast-induced nephropathy (CIN) is increasingly seen in patients receiving contrast medium. Abelmoschus manihot (L.) Medik. (Malvaceae) and its preparations are widely used and effective in the treatment of various chronic kidney diseases and CIN in China. It is supposed to be an important adjuvant therapy for CIN.

**Methods:**

PubMed and CNKI were searched for the main compounds of *A. manihot* L. The Swiss target prediction platform, OMIM, GeneCards, DisGeNET, and DrugBank databases were mined for information relevant to the prediction of targets that *A. manihot* L. in the treatment of CIN. Subsequently, STRING database was applied for the construction of the PPI protein interaction network, meanwhile, the core targets were screened. DAVID database was used to perform the GO function and Kegg signal pathway enrichment analysis. AutoDockTools and PYMOAL were used for molecular docking. Vitro experiments were used to verify the effect of TFA, the main active component of *A. manihot* L., in the intervention of iopromide-induced cells injury.

**Results:**

A total of 17 chemical components and 133 potential targets in *A. manihot* L. were obtained. The top 15 proteins with higher degree value were selected from the PPI network model, AKT1, PIK3R1, EGFR, SRC,AR, APP, TNF, GAPDH, MMP9, and PTPN1, etc. may be core targets. The enrichment analysis indicated that *A. manihot* L. was involved in the regulation of PI3K/AKT signaling pathway, FoxO signaling pathway, VEGF signaling pathway, HIF-1, TNF signaling pathway, melanoma, hepatitis B, and other signaling pathways which were mainly associated with the regulation of transcription and apoptosis, protein phosphorylation, inflammatory response, aging, and cell proliferation. Molecular docking indicated that the key components and core targets had a good binding ability. The vitro experiments illustrated that TFA reduces iopromide induced renal tubular cell injury and apoptosis, which may be related to regulating the phosphorylation of AKT.

**Conclusion:**

The study preliminarily revealed the multi-component, multi-target, and multi-pathway synergistic effects of *A. manihot* L. on CIN, which provide theoretical reference and basis for the study of the pharmacological mechanism of *A. manihot* L. in the treatment of CIN.

## Introduction

Contrast-induced nephropathy (CIN) is a sudden deterioration of renal function caused by intravascular injection of contrast medium (CM) for diagnosis and interventional operation. It is generally defined as renal function damage within 3 days after intravascular injection of CM without other causes, and serum creatinine (Scr) increases by more than 25% or 44μmol/L compared with baseline levels (1). With the wide application of CM in the field of radiation diagnosis and interventional therapy, the incidence of CIN is increasing year by year. It is a common complication of various ductus arteriosus diagnosis and treatment techniques represented by the percutaneous coronary intervention (PCI), and it is also a representative sign of poor patient prognosis. The incidence of CIN is estimated to range from 3.3% to 10.5% but can be as high as 10% to 20% or even 50% in high-risk patients (2, 3). Contrast-induced acute kidney injury (CI-AKI) is currently the third leading cause of hospital-acquired AKI (4, 5). The occurrence of CIN is related to a variety of factors, age, diabetes, renal insufficiency, progressive heart failure, low ejection fraction, acute myocardial infarction, cardiogenic shock, and kidney transplantation are fixed risk factors for CIN. Contrast agent dose, hypotension, anemia, and blood loss, dehydration, hypoalbuminemia, ACEI, diuretics, NSAIDs, nephrotoxic antibiotics, intra-aortic balloon counterpulsation are reversible risk factors for CIN (6, 7). Studies have shown that CIN increases the risk of major adverse events such as renal failure and cardiovascular events, such as acute myocardial infarction, stroke, and end-stage renal disease requiring kidney replacement (8–10). Current guidelines do not support pharmacological interventions to prevent CIN because none of the pharmacological practices has been shown to provide consistent protection (1). To date, there are no recognized effective preventive measures. At present, the prevention and treatment of CIN are mainly to monitor renal function, avoid the combined use of nephrotoxic drugs, maintain effective blood volume, postoperative hydration, and other non-specific treatments (11, 12). CIN is the culprit that leads to the rapid deterioration of renal function, and it is a sign of increased short-term and long-term cardiovascular events and adverse renal events in patients. It is critical for early intensive intervention in high-risk patients with CIN.

Traditional Chinese Medicine (TCM) is the most common form of alternative medicine and supplementary medicine in Asia, and has been widely used in the treatment of CKD. Abelmoschus manihot (L.) Medik. (Malvaceae) is a herb used in Traditional Chinese Medicine to treat some kidney diseases. Modern pharmacology has confirmed that the flavonoids of Abelmoschus manihot L. have the effects of anti-inflammation, antipyretic, analgesia, relieving cardio-cerebral myocardial ischemia, and hypoxia, protecting renal tubules and glomeruli (13). *A. manihot* L. and its preparations are widely used and effective in the treatment of various chronic kidney diseases and CIN, but the pharmacological mechanism of its treatment of CIN has not been fully elucidated. Therefore, this study undertook an in-depth analysis and prediction of the active component-target-pathway of Abelmoschus manihot L. in the treatment of CIN by the method of network pharmacology and explores the mechanism of *A. manihot* L. in the treatment of CIN.

## Materials and Methods

### Collection of Active Components From *A. manihot* L.

All the chemical constituents of *A. manihot* L. were searched in CNKI and PubMed. The chemical constituents with pharmacological activities were selected and input into the PubChem database (https://pubchem.ncbi.nlm.nih.gov/) to query the serial number of CAS and download the corresponding sdf molecular structures of the active components. The main active components of *A. manihot* L. were determined.

### Target Prediction of the Main Active Components

The sdf molecular structures of active components were imported into the Swiss Target Prediction platform (14). The species was limited to “Homo Sapiens”. Active components were subject to target enrichment prediction and the predicted genes were collected. All the genes were integrated and duplicates were removed. These genes constitute the prediction targets of the main active components of *A. manihot* L.

### Collection of Targets Related to CIN

A search for target genes related to CIN was performed in the OMIM database (https://www.omim.org/), Gene cards database (https://www.genecards.org/), DrugBank database (https://www.drugbank.ca/), DisGeNET database (https://www.disgenet.org/) with the keywords “Contrast Induced Nephropathy” and “CIN”. Duplicates were deleted and the main genes related to CIN were integrated for further research.

### Acquiring Targets of Main Active Components for CIN Treatment

Venny 2.1.0 (http://bioinfogp.cnb.csic.es/tools/venny/index.html) software was used to obtain the intersection between the predicted targets of *A. manihot* L. and the main targets of CIN. A Venn diagram was drawn. These intersection genes may be the related targets of *A. manihot* L. in the treatment of CIN.

### Construction of Protein-Protein Interaction (PPI) Network and Screening of the Core Genes

The intersection of the predicted targets in the network model were imported into the String11.0 database (https://string-db.org/) (15), the species was limited to “Homo Sapiens”. The highest confidence protein score value was >0.7. Unconnected single proteins were removed to construct the PPI diagram of these core targets. Then the relevant information of the PPI diagram is imported into the Cytoscape 3.6.1 software to construct a network model and perform topological analysis, and the node size, color, and edge thickness are adjusted according to the degree and comprehensive score. Taking the value as a reference, the core protein targets in the PPI network were screened.

### Enrichment Analysis of Gene Ontology (GO) and Signaling Pathway

The core protein targets were Imported into DAVID online analysis platform (https://david.ncifcrf.gov/) (16) for enrichment analysis of the biological process (BP), molecular function (MF), and cellular component (CC). The Kyoto Encyclopedia of Genes and Genomes (KEGG) databases were used to enrichment analyze the signaling pathways related to the targets. The species and background were both limited to “Homo Sapiens”. The main BPs, MFs, CCs, and pathways of *A. manihot* L. for CIN treatment were selected according to the criterion of *p*<0.01. The smaller the *p*-value, the higher the degree of enrichment. The top 20 GO biological functions were selected. The network model was established and the bar chart was drawn. Based on the results of BPs and signaling pathways related to the targets enriched in the GO-BP and KEGG database, the enrichment results were visualized and the bubble diagram was drawn.

### Construction and Analysis of the Network Model of Main Active Components-CIN Treatment Related Targets-CIN Treatment Related Signaling Pathways

The main active components, the corresponding CIN related targets, and CIN treatment related signaling pathways were imported into Cytoscape 3.6.1 software. The main active component-target-signaling pathways network model was drawn. The topological analysis was used to analyze the network model and calculate the degree. The larger the value of the degree, the more important the node is in the network. The node size, color, and edge thickness are adjusted according to the degree, and visualization was carried out.

### Molecular Docking Verification

Molecular docking was carried out between the key compounds of *A. manihot* L. and the core targets obtained from the PPI network. The receptor protein encoded by the gene was identified by the UniProt database (https://www.uniprot.org/), and its three-dimensional structure was obtained from the RCSB PDB database (https://www.rcsb.org/). The PubChem database (https://pubchem.ncbi.nlm.nih.gov/) was used to download the two-dimensional structure of the molecular ligand. Then the structures were imported into the PyMOL2.4.0 software for receptor protein dehydration. The hydrogenation and charge calculation of the protein was carried out by AutoDockTool for structure optimization. The parameters of the receptor protein docking site were set to include active pocket sites that bind to small molecules of ligands. Finally, the AutoDockTools-1.5.6 software was used to dock the key active components with the corresponding targets, optimize the binding affinity between receptors and ligands and obtain the three-dimensional structures.

### Preparation of Total Flavonoids of *A. manihot* L.

Total flavonoids of *A. manihot* L. (TFA) were extracted from the dry corolla of *A. manihot* L. TFA was extracted by the Department of Drug Preparation of the Affiliated Hospital of Nanjing University of Chinese Medicine. Briefly, 0.5 kg of raw *A. manihot* L. flowers was immersed in 8000mL 75% ethanol for 1 h and then heated to 90°C for 1 h to achieve alcohol extraction as ambrette fluid extract. After filtration, the extract was evaporated to produce a dry extract powder under vacuum at 60°C. The dried residue was dissolved in water for use in the experiments (17).

### Experimental Verification

The data of network pharmacology show that the flavonoids are the key components of *A. manihot* L. in the treatment of CIN, and AKT is the core target. TFA inhibits the progression of Contrast-induced Nephropathy mainly by regulating apoptosis. Previous studies have successfully established the injury model of renal tubular epithelial cells (HK2) with iopromide. Iopromide can induce apoptosis of HK2 cells. Therefore, we used iopromide to establish an *in vitro* model of renal injury induced by a contrast medium. Then TFA was used to interfere with the model to evaluate whether it can regulate AKT phosphorylation and reduce apoptosis.

### Reagents

Bcl-2 (lot no. AF6139) and Bax (lot no. AF0120) were purchased from Affinity Biosciences Co., LLC. (Cincinnati, USA). Caspase3 (lot no. 9662) and Cleaved Caspase-3 (Asp175) Antibody (lot no. 9661) were purchased from Cell Signaling Technology Co., Ltd. (Shanghai, China). Antibodies to AKT (lot no. 60203-2-Ig), antibody to phospho-AKT (Ser473)(lot no. 66444-1-Ig), antibody to GAPDH (lot no. 60004-1-Ig), horseradish peroxidase (HRP)-conjugated Affinipure goat anti-mouse IgG (H+L)(lot no. SA00001-1) and HRP-conjugated Affinipure goat anti-rabbit IgG (H+L) (lot no. SA00001-2) were purchased from Proteintech Biotechnology Co., Ltd. (Wuhan, China). Iopromide was purchased from Bayer Co., LLC. (Leverkusen, Germany). Fetal bovine serum (FBS), 0.25% trypsin-EDTA, phosphate buffered saline (PBS), and Dulbecco’s modified Eagle medium/F-12 (DMEM/F-12) were purchased from Thermo Fisher Scientific (Scoresby, Australia). Cell Counting Kit-8 (CCK-8) was obtained from APExBIO (Houston, TX, USA). One Step TdT-Mediated dUTP Nick End Labeling (TUNEL) Apoptosis Assay Kit was purchased from Vazyme Biotechnology Co., Ltd. (Nanjing, Jiangsu). 4,6-diamidino-2-phenylindole (DAPI) staining solution and BCA protein assay kit were purchased from Beyotime Biotechnology (Shanghai, China).

### Cell Line and Culture

Normal renal tubular epithelial cells (HK2) were obtained from Saiku Biotechnology Co., Ltd. The cells were maintained in DMEM/F-12 supplemented with 10% FBS and incubated at 37°C in 5% CO_2_. The cells at approximately 80% to 90% confluency were digested and passaged.

### Cellular Viability

HK2 cells were digested and seeded in 96-well plates at 1×10^4^ cells per well. After 24 h of incubation, the cells were cultured in a serum-free medium and stimulated with iopromide (111mg I/mL) in the absence and presence of TFA (0.6 mg/mL). After 12 h of treatment, the medium was discarded, and 100μL serum-free medium containing 10% CCK-8 was added to each well. After incubating for another 1 h, the absorbance was measured using a model ELx800 spectrometer (BioTek Instruments, Winooski, VT, USA) at a wavelength of 450 nm. Cell viability is expressed as a percentage of the control group.

### Fluorescence Staining of Apoptotic Cells

TUNEL staining kit and DAPI staining solution were used to assess the presence of apoptotic cells. Briefly, the cells were incubated and treated in accordance with the experimental protocol, washed three times with PBS, and fixed with 4% paraformaldehyde for 30 min. Then, they were permeabilized with 0.3% Triton X-100 in PBS for another 30 min and washed three times with PBS. 50μL of TUNEL staining solution was added to each well and incubated in the dark for 1 h at 37°C. Then, cells were washed three times with PBS, and 100μL of DAPI staining solution was added to each well, followed by incubation in the dark for 10 min and washing with PBS. Finally, cell fluorescent images were captured using a fluorescence microscope (Nikon Ni-U, DS-Qi2) equipped with standard red and ultraviolet fluorescence cubes. Fluorescent images were analyzed quantitatively using ImageJ software.

### Western Blot Analysis

After incubation and intervention, the cells were lysed in RIPA buffer supplemented with a 2% protease and phosphatase inhibitor cocktail for 20 min on ice. After sonication, the samples were centrifuged at 4°C for 20 min, and each supernatant was collected for subsequent experiments. Total protein concentration was determined using a BCA protein assay kit. Equal amounts of protein (20µg) were separated by 10% or 12% sodium-dodecyl sulfate-polyacrylamide gel electrophoresis (SDS-PAGE). The resolved proteins were electrophoretically transferred to polyvinylidene difluoride 9 (PVDF) membranes (Millipore, Billerica, MA, USA). The membranes were blocked with 5% non-fat dried milk in PBS containing Tween (PBST) buffer for 1 h at room temperature, washed three times with PBST, and incubated with primary antibodies overnight at 4°C. The membranes were washed again and incubated with HRP-conjugated anti-rabbit or anti-mouse IgG for 1 h at room temperature. The bands were flushed with chemiluminescent HRP substrate and imaged using a chemiluminescence system (ChemiDocTM XRS+ from Bio-Rad, CA, USA). Western blot images were analyzed quantitatively by Image Lab software. GAPDH was assayed to confirm equal loading of proteins.

### Statistical Analyses

Statistical analyses were performed using SPSS software. All values were expressed as mean ± standard deviation (SD). Comparisons of two populations were made using an unpaired 2-tailed Student’s t-test. For multiple groups, statistical significance was determined using a one-way analysis of variance (ANOVA) followed by Dunnett’s test. Statistical significance was set at *p*<0.05.

## Results

### Main Active Components of *A. manihot* L.

Through the collation of related papers (13, 18–23), a total of 17 chemical components such as flavonoids, organic acids and steroids in *A. manihot* L. were obtained as the main active components of study. The specific information is shown in **Table 1**.

**Table 1:** Main active components of *A. manihot L.*.

Category	Molecule Name	CAS	Sdf
**Flavonoids**	Quercetin	117-39-5	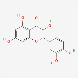	
	Rutin	153-18-4	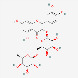	
	Hyperoside	482-36-0	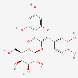	
	Isoquercetin	482-35-9	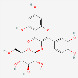	
	Quercetin 3’-O-glucoside	19254-30-9	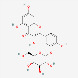	
	Quercimeritrin	491-50-9	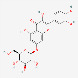	
	Gossypetin	489-35-0	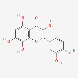	
	Myricetin	529-44-2	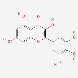	
	Myricetin-3-O-β-glucoside	19833-12-6	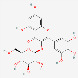	
**Organic acids**	Caffeic acid	501-16-6	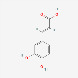	
	Gallic acid	149-91-7	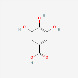	
	Protocatechuic acid	99-50-3	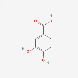	
	2,4-dihydroxybenzoic acid	89-86-1	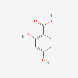	
**Steroids**	β-sitosterol	64997-52-0	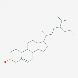	
	Sitgmasterol	83-48-7	
	α-spinasterol	481-18-5	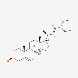	
**Nucleosides**	Adenosine	58-61-7	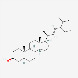	

### Target Prediction of *A. manihot* L.

The sdf molecular structures of all the main active components in **Table 1** were imported into the Swiss Target Prediction platform, and the species was set up to “Homo Sapiens”. The related targets of the above active components were predicted, all the information of targets was collected, and a total of 133 potential targets of *A. manihot* L. were obtained after deleting the integration of repeated invalid targets.

### Related Targets of CIN

Search OMIM database, Genecards database, DrugBank database, and DisGeNET database with “Contrast Induced Nephropathy” and “CIN” as keywords. A total of 2832 CIN related targets were screened.

### Key Targets of *A. manihot* L. in Treatment of CIN

**Figure 1:**
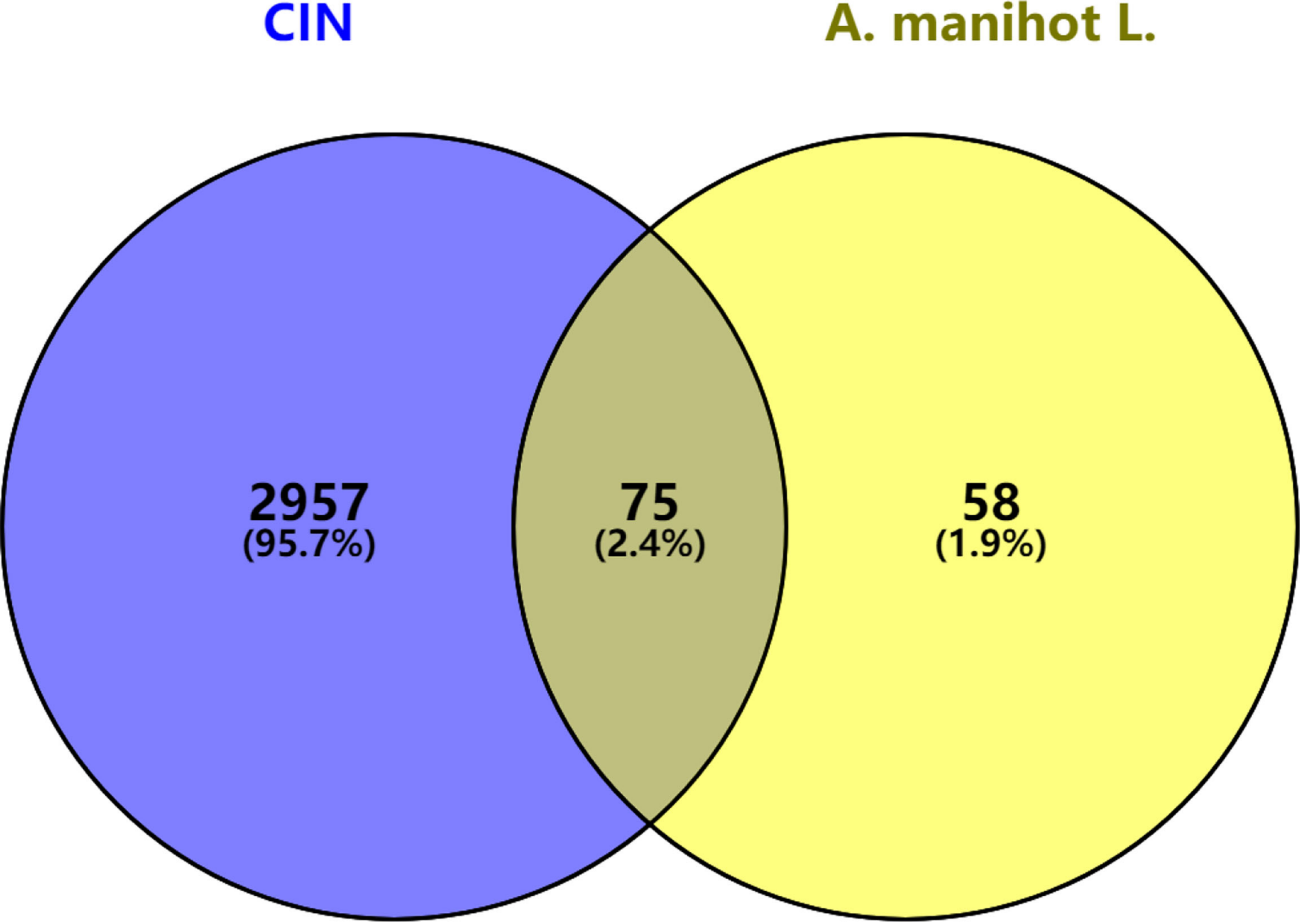
Intersection targets of *A. manihot L.* in the treatment of CIN.

The predicted related targets of *A. manihot* L. were mapped to the main targets related to CIN using Venny 2.1.0 software. A Venn diagram was drawn (**Figure 1**). A total of 75 intersection targets were obtained. These may be the core targets of *A. manihot* L. for CIN treatment.

### Construction of PPI Network Model

**Figure 2:**
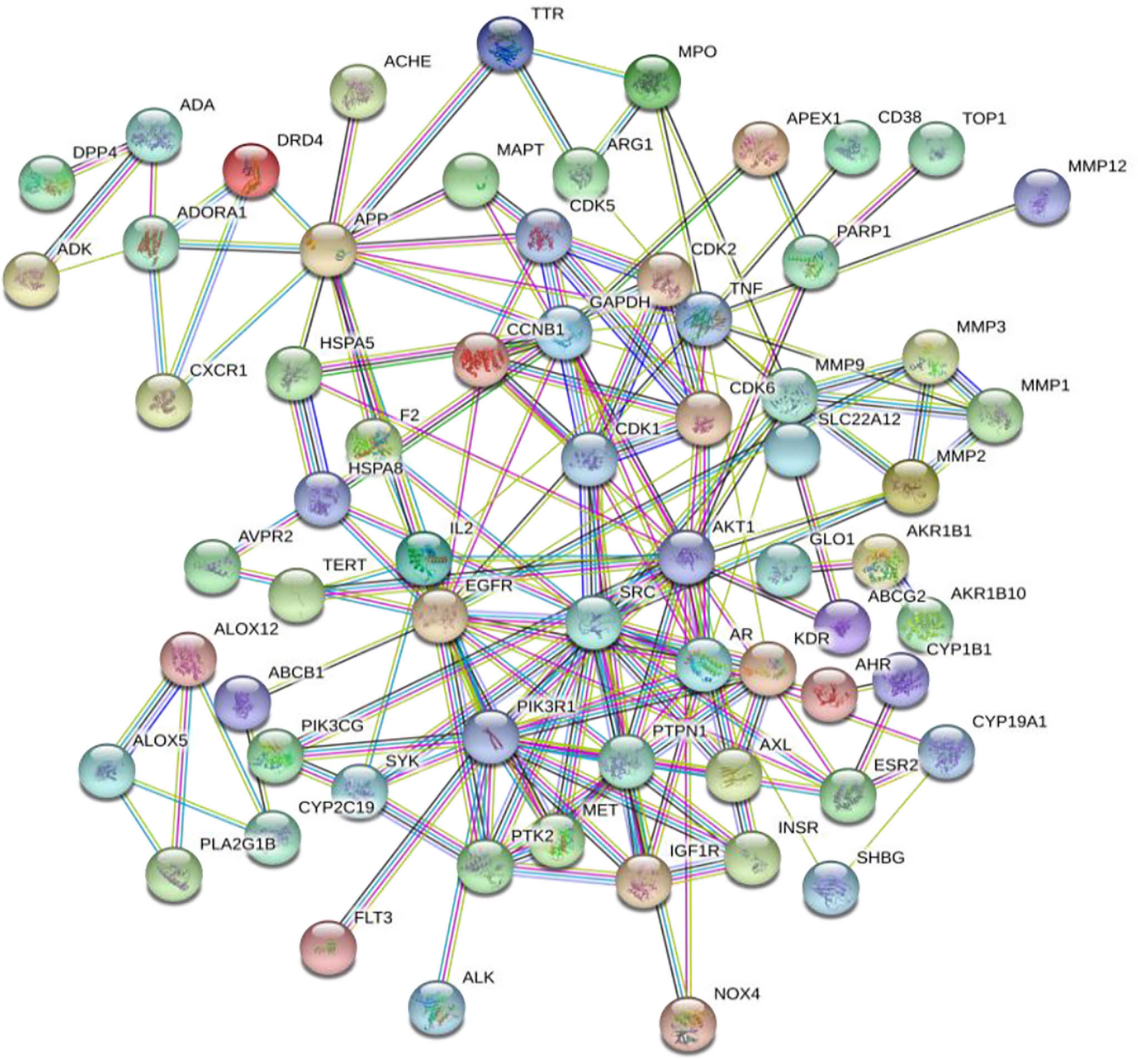
The PPI network model.

**Figure 3:**
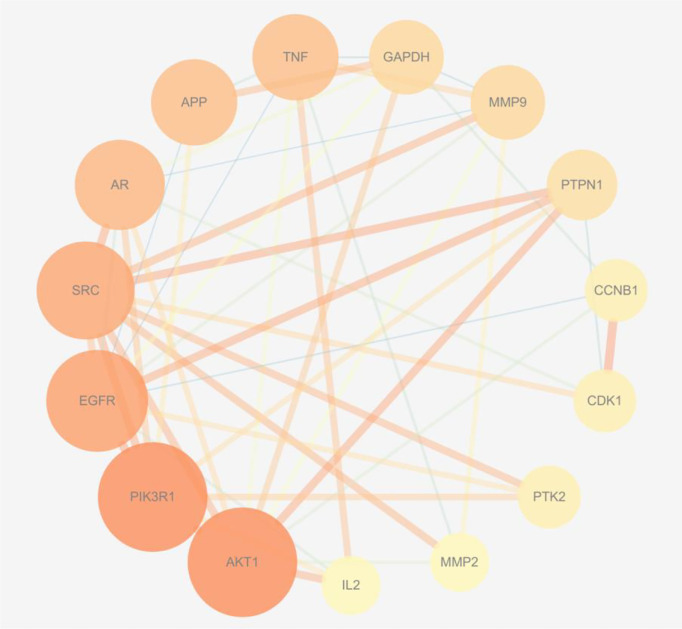
The core targets in the PPI network. The bigger the node, the larger the degree value. The thicker the edge, the closer the relationship between the proteins. The darker the color, the more important the target.

The 75 intersection targets of *A. manihot* L. in treatment of CIN were imported into the String database, and the species was limited to “Homo sapiens”. The relationship diagram of protein-protein interaction was obtained, free targets were excluded, the highest confidence protein score value was set as > 0.7, a PPI network was constructed (**Figure 2**). The relevant information was obtained and imported into the Cytoscape software. The top 15 proteins with higher degree value were selected (**Figure 3**), including AKT1, Phosphatidylinositol 3-kinase regulatory subunit alpha (PIK3R1), epidermal growth factor receptor (EGFR), SRC, Androgen receptor (AR), Amyloid-beta A4 protein (APP), Tumor necrosis factor (TNF), Glyceraldehyde-3-phosphate dehydrogenase (GAPDH), Matrix metalloproteinase-9 (MMP9), Tyrosine-protein phosphatase non-receptor type 1 (PTPN1), and others. The PPI network was visualized and analyzed. The node color and size have been adjusted according to the degree, the bigger the node, the larger the degree value. The thicker the edge, the closer the relationship between the proteins. The darker the color, the more important the target. These targets may be core targets of *A. manihot* L. in the treatment of CIN. The targets are involved in the regulation of cell proliferation and apoptosis, metabolism, growth, and angiogenesis, and are closely related to the development and prognosis of CIN. It is suggested that the above targets may play a crucial role in the treatment of CIN. These findings confirmed the multi-component and multi-target synergistic mechanism of *A. manihot* L. in the treatment of CIN.

### Enriched Gene Biological Functions and Signaling Pathways

The 75 related core targets were uploaded to the DAVID database for GO enrichment analysis and KEGG pathway enrichment analysis. A *p ≤* 0.01 was the screening parameter. 157 biological processes (BP), 40 cellular composition (CC), 36 molecular functions (MF), and 73 KEGG signaling pathways were obtained. GO and KEGG enrichment analyses were carried out to explore the potential function and mechanism of target genes related to the treatment of CIN in *A. manihot* L.

**Figure 4:**
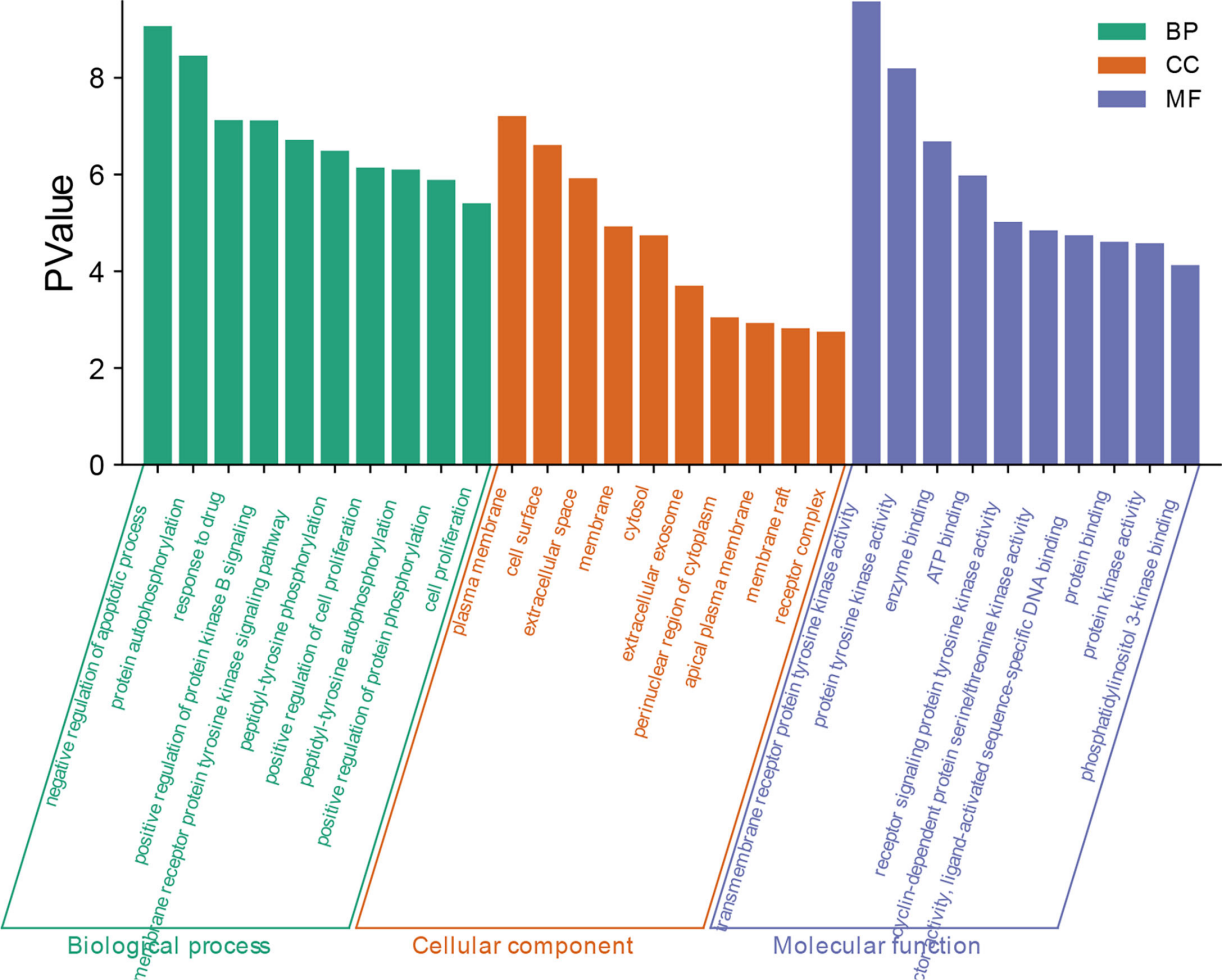
Enrichment analysis of gene biological functions.

**Figure 5:**
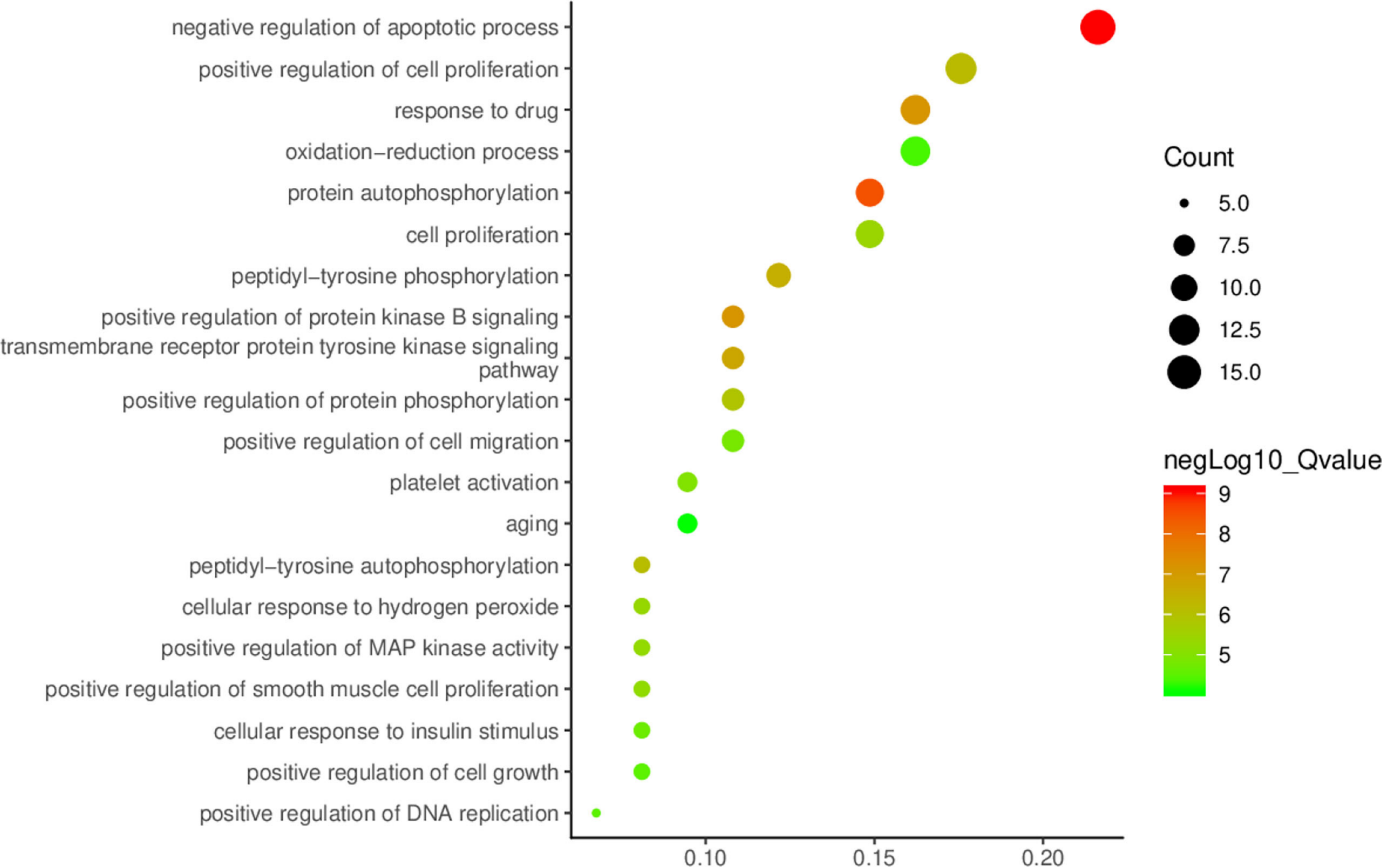
Enrichment analysis of biological process.

**Figure 6:**
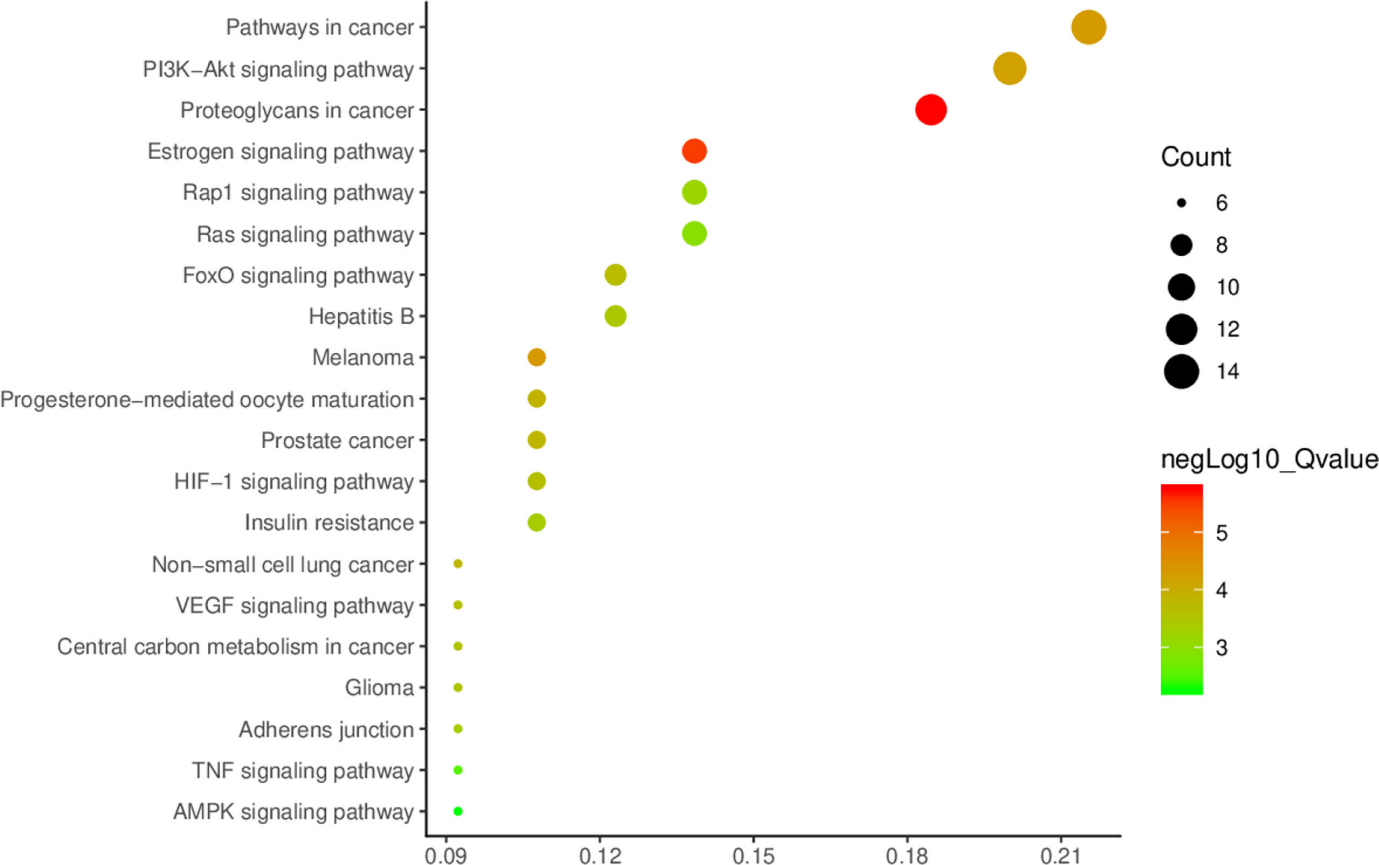
Enrichment analysis of KEGG signaling pathway.

With *p*<0.01 as the screening criterion, the first 10 GO biological functions of these targets were shown in the form of a histogram (**Figure 4**). The top 20 BPs and KEGG signaling pathways enriched were plotted as bubble diagrams (**Figures 5**, **6**). In the bubble diagram, the node size represents the gene number, the larger the node, the more genes corresponding to the node. The color of the node represents the *p*-value. The smaller the *p*-value, the redder the color, otherwise, the values were depicted in shades of green. BPs enrichment analysis showed that these targets were involved in the composition of a variety of cell components and regulated the activities of a variety of kinases and transcription factors. The therapeutic effect of *A. manihot* L. on CIN may be related to the negative regulation of apoptosis, protein autophosphorylation, positive regulation of protein kinase B signal transduction, transmembrane receptor protein tyrosine kinase signaling pathway, cell proliferation, oxidative stress, MAPK activation and so on. The CCs may have therapeutic roles in CIN by affecting BPs, such as the regulation of transcription and apoptosis, protein phosphorylation, inflammatory response, aging, and cell proliferation.

KEGG signaling pathway enrichment analysis showed that *A. manihot* L. was involved in the regulation of PI3K/AKT signaling pathway, FoxO signaling pathway, VEGF signaling pathway, HIF-1, TNF signaling pathway, melanoma, hepatitis B, and other signaling pathways, which were closely related to the pathogenesis of CIN.

### Construction and Analysis of the Network Model of Main Active Components-CIN Therapy Related Targets-CIN Therapy Related Signaling Pathways

**Figure 7:**
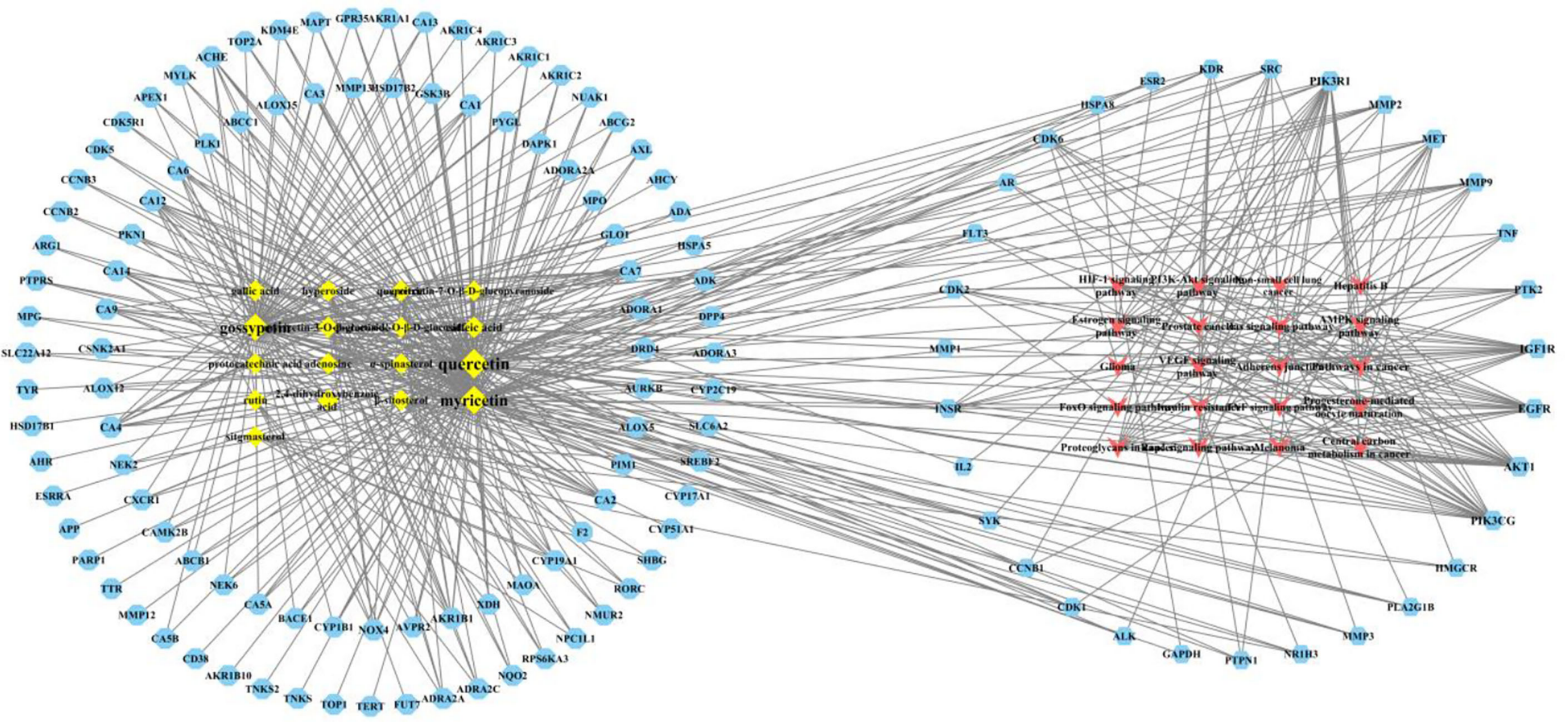
Main Active Components-Targets-Signaling Pathways network model.

The main active components, CIN therapy related targets, and CIN therapy related signaling pathways were imported into Cytoscape 3.6.1 to build a main active component-target-signaling pathway network model (**Figure 7**). The topology analysis of the network model is carried out, the model included 170 nodes and 555 edges. In this model, 17 active components were shown as diamond-shaped yellow nodes, 133 targets were exhibited as blue regular hexagon nodes, 20 signaling pathways were marked as a red triangle. The edges represent the interaction between the components and the targets. The network model shows the characteristics of cooperative regulation of multi-components, multi-targets, and multi-pathways in the treatment of *A. manihot* L. We found that the PI3K/AKT signaling pathway, FoxO signaling pathway, VEGF signaling pathway, HIF-1 signaling pathway, and TNF signaling pathway are important signaling pathways in the treatment of CIN. It is worth noting that most of the core targets in the PPI network participate in the PI3K/AKT signaling pathway, and the results are consistent with the results of KEGG enrichment. Therefore, we chose the PI3K/AKT signaling pathway for further exploration to determine the potential mechanism of *A. manihot* L. for CIN treatment.

### Docking of Core Components and Corresponding Main Targets of *A. manihot* L.

**Figure 8:**
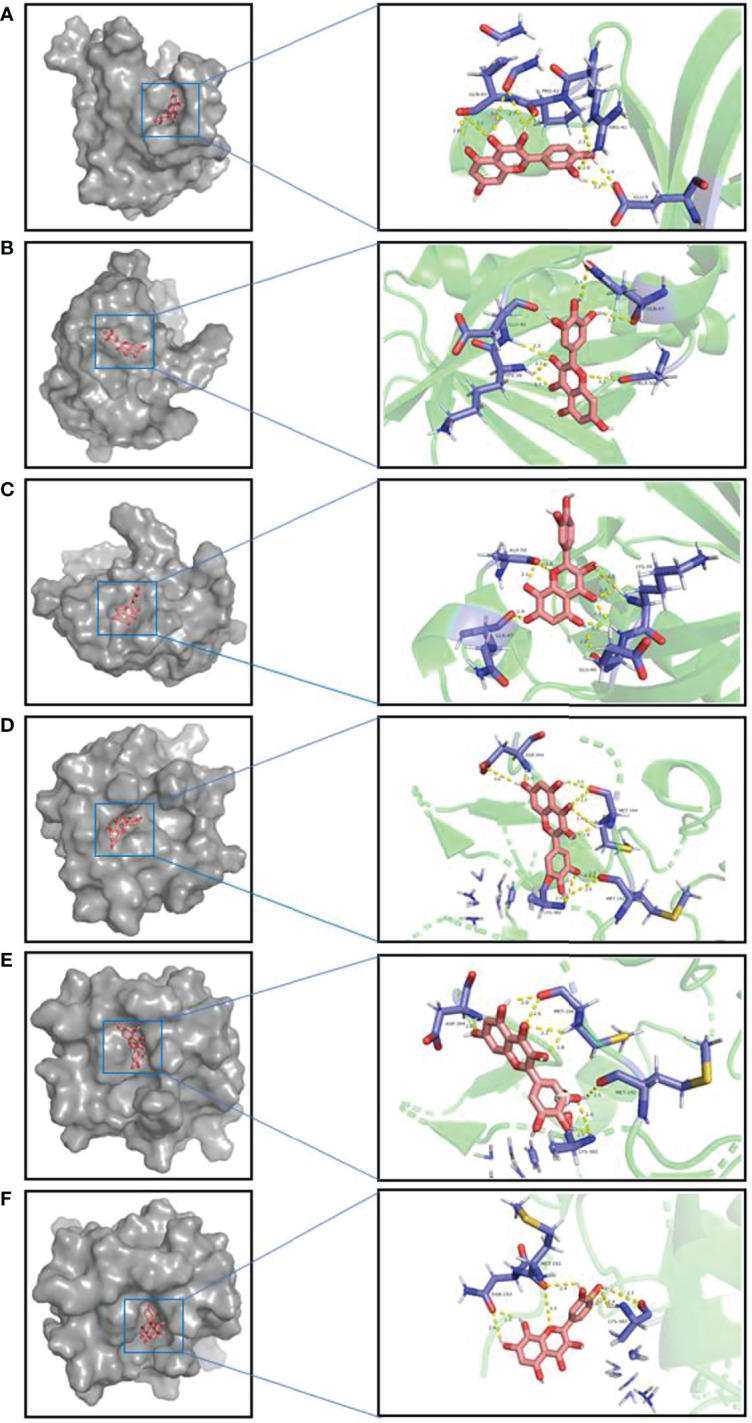
Schematic diagram of molecular docking **(A)**. AKT1-Quercetin. **(B)** AKT1-Myricetin. **(C)** AKT1-Gossypetin. **(D)** PIK3R1-Quercetin. **(E)** PIK3R1-Myricetin. **(F)** PIK3R1-Gossypetin. Interacting amino acids and compound structures are shown on lines, in which hydrogen bonds are shown in yellow, amino acids are shown in purple, compound structures are shown in pink.

The top three components (Quercetin, Myricetin, Gossypetin) selected from the 17 key components were docked with the corresponding two targets (AKT1,PIK3R1) of the 15 core targets. The molecular docking showed that all these active compounds could easily enter and bind to the active pockets of pathway-related proteins. The binding affinity < 0 indicates that the receptor and the ligand have the ability of spontaneous binding. The lower the binding affinity score is, the more stable the crystal structure of the protein corresponding to the formation of the compound and the target is. The results of molecular docking show that the binding affinity between the key components and corresponding core targets of *A. manihot* L. did not exceed -4.5 kcal/mol, which indicated that the key components and core targets had a good binding ability (**Figure 8**, **Table 2**). The main active components of *A. manihot* L. may play a therapeutic role in CIN by intervening in the corresponding core targets.

**Table 2:** The binding affinity between components and targets in molecular docking.

Component	Target	Binding Affinity (kcal/mol)
Quercetin	AKT1	-5.47
	PIK3R1	-8.26
Myricetin	AKT1	-4.78
	PIK3R1	-7.57
Gossypetin	AKT1	-5.25
	PIK3R1	-6.41

### TFA Reduces Iopromide Induced Renal Tubular Cell Injury and Apoptosis by Regulating the Phosphorylation of AKT

**Figure 9:**
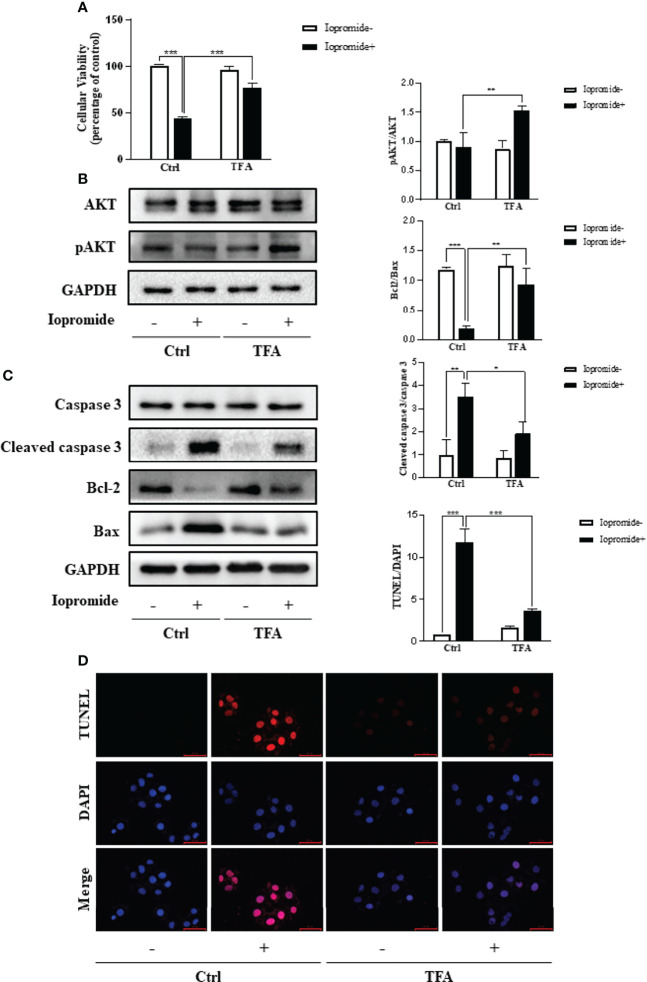
TFA Reduces Iopromide induced Renal Tubular Cell Injury and Apoptosis by Regulating the Phosphorylation of AKT. **(A)** Effects of TFA on cellular viability of iopromide induced cell iniury. HK2 cells in 96-well plates were pretreated with TFA (0.6mg/mL) for 1 h and then challenged with or without iopromide (111mg I/mL) for another 12 h. Cellular viability was evaluated using a CCK-8 assay. Data are expressed as the percentages of living cells versus the control (Ctrl) (means ± SD, n=3, ***p<0.001 versus in Ctrl). **(B)** Effects on the phosphorylation of AKT in iopromide induced cells injury. HK2 cells were exposed to the indicated concentrations of iopromide with or without TFA for 12 h. Cellular lysates were subjected to western blot analysis of AKT, phosphorylated AKT and GAPDH. Statistical analysis opf phosphorylated AKT is shown on the right (means ± SD, n=3, **P<0.01 versus in Ctrl). **(C)** Effects of TFA on expressioin of apoptosis related proteins. HK2 cells werte pretreatred with TFA for 1 h and then challenged with or without iopromide for another 12 h. Cellular lysates were subjected to western blot analysis of cleaved caspase3, Bcl-2 and Bax. Statistical analysis of results are shown on the right (means ± SD, n=3, *P<0.05, **P<0.01, ***P<0.001). **(D)** Effects of TFA in iopromide induced cells apoptosis by TUNEL staining assay. HK2 cells were incubated with TFA for 12 h in the presence and absence of iopromide. Apoptosis cells were detected by TUNEL staining. Flourescence staining was observed by flourescence microscope (magnification x 200). Statistical analysis of results are shown on the right (means ± SD, n=3, ***P<0.001).

To further observe the effect of CM on renal tubular epithelial cells, we established an *in vitro* model of CIN induced by iopromide. TFA was used to intervene iopromide-induced HK2 cells injury, and its effect on cellular viability was evaluated by the CCK8 method. Compared with the iopromide group (111mgI/mL), the TFA group (0.6mg/mL) significantly increased cell viability (**Figure 9A**), so TFA could reduce the cell injury induced by iopromide. The results of network pharmacology show that PI3K/AKT signaling pathway may be an indispensable pathway for *A. manihot L.* in the treatment of CIN. AKT is widely involved in a variety of physiological processes *in vivo*. AKT, as the core target of PPI network enrichment, has a good binding ability with the core components of *A. manihot* L. We found that TFA can significantly increase the phosphorylation level of AKT (**Figure 9B**), which indicates that AKT may be an important target for TFA to reduce the injury of renal tubular epithelial cells induced by iopromide. Meanwhile, TFA attenuated iopromide-induced apoptosis in HK2 cells, which was confirmed by TUNEL staining (**Figure 9D**). We further tested the expression of apoptosis-related proteins including Caspase3, Bcl2, and Bax (**Figure 9C**), and the results of Western blot confirmed the above conclusion.

## Discussion

Intravascular injection of CM is an irreplaceable step in percutaneous coronary intervention. With the wider application of CM, CIN has become a common complication after PCI. CIN has a significant negative impact on public health and finances and CIN-related diseases are a major healthcare problem. In the US healthcare system, more than 2 million cardiac interventional operations were performed each year, and more than 30 million doses of the iodine CM were used (24).

The pathophysiological mechanism of CIN is complex, and its explicit mechanism is far from clear, especially its cellular and molecular mechanism (25). The CM has a direct cytotoxic effect on vascular endothelial cells and renal tubular epithelial cells. After intravascular injection of CM, changes in renal hemodynamics occurred, characterized by rapid and transient vasodilation, accompanied by continuous vasoconstriction, increased renal vascular resistance, and decreased renal blood flow, resulting in a decrease in glomerular filtration rate and renal ischemia (26, 27). In addition, CM can cause osmotic diuresis, increase fluid excretion, increase renal tubular reabsorption, vasoconstriction is magnified by secondary renal insufficiency, resulting in a decrease in renal blood flow and oxygen supply, an increase in oxygen consumption and aggravation of ischemic and anoxic symptoms of the medulla (28), which eventually leads to renal tubule injury and tubule formation (29). Hypoxia induces the formation of reactive oxygen species (ROS) (30). A great deal of evidence shows that ROS plays an important role in the renal damage caused by CM (31, 32). ROS can directly damage renal tubular epithelial cells and vascular endothelial cells. CM increases the production of ROS and oxidative stress in kidneys and mediates cell membrane damage, which leads to apoptosis and necrosis, especially in renal tubular epithelial cells in the outer medulla (33, 34). CM induces apoptosis mainly through internal or mitochondrial cleavage of caspase-3 and caspase-9, these proteases are the executors of cutting key cellular proteins (35). This apoptotic pathway is regulated by members of the Bcl-2 family (36, 37).

Studies have shown that *A. manihot* L. can improve renal function, such as improving podocyte apoptosis, inhibiting immune response, reducing inflammation, improving renal fibrosis, and reducing renal injury (38, 39). Meanwhile, *A. manihot* L. has the effect of scavenging oxygen free radicals (40), which is helpful to improve the oxidative stress damage caused by CIN. Clinical randomized controlled trials show that perioperative prophylactic use of *A. manihot* L. and its pharmaceutical preparation during the percutaneous coronary intervention can effectively reduce the incidence of CIN (41, 42). Network pharmacology is an effective method to explore the relationship between plant medicine, targets, and diseases. at present, the network pharmacological analysis has been widely used to study the pharmacological mechanism of traditional Chinese medicine. *A. manihot* L. contains quercetin, rutin, myricetin, hyperin, and other 17 active components in the potential treatment of CIN, which are numerous and complex. This method is used to predict the mechanism of *A. manihot* L. in the treatment of CIN. Our study provides evidence that *A. manihot* L. ameliorates iopromide-induced CIN, which may be mediated by the activation of the PI3K/AKT signaling pathway.

The results of network pharmacology show that many pathways are closely related to the pathogenesis of CIN. PI3K/AKT, FoxO, VEGF, HIF-1, TNF, and other signaling pathways are vital pathways. The PI3K/AKT signaling pathway is related to cell growth and proliferation. FOXO is a key downstream factor of the PI3K/Akt signaling pathway, which induces the expression of death receptor ligands and Bcl-2 family members through negative regulation of the PI3K-Akt signaling pathway, and participates in cell survival, proliferation, growth, and angiogenesis (43, 44). Apoptosis signaling transduction plays an important role in the pathogenesis of CIN. Studies have confirmed the protective role of phosphorylation of AKT in the process of cell injury (45). Regulation of the PI3K/AKT signaling pathway can down-regulate cleaved caspase-3 and reduce the ratio of Bax/Bcl2, which can reduce apoptosis induced by CM (46).

Molecular docking was used to verify the binding ability of the main active components of *A. manihot* L. and its potential therapeutic targets. The results illuminated that there was a strong interaction between the active components and the proteins of the PI3K/AKT signaling pathway, which further verified the therapeutic effect of the main active components of *A. manihot* L. on CIN. We selected the TFA to verify the protective mechanism *in vitro*. By regulating the phosphorylation of AKT, TFA significantly reduced iopromide-induced apoptosis in HK2 cells. These results suggest that the treatment of *A. manihot* L. on CIN may be mediated by the regulation of the PI3K/AKT signaling pathway.

This study is a preliminary attempt to explore the effect of active components of *A. manihot* L. on the PI3K/AKT signaling pathway. It may provide a new direction for further study on the molecular mechanism of active compounds interfering with CIN. Nevertheless, there are some limitations to the study. The network pharmacology, which was based on the information collected in various databases, challenges the reliability of data collection and the accuracy of conclusions. Considering the importance of the PI3K/AKT signaling pathway in this network pharmacology, our experiment is limited to paying attention to the PI3K/AKT signaling pathway, and other pathways need to be further verified.

## Conclusion

In this study, network pharmacology was used to explore the potential mechanism of *A. manihot* L. on CIN, which may be related to the regulation of the PI3K/AKT signaling pathway. Studies have shown that flavonoids are important active components of *A. manihot L.* in the treatment of CIN. AKT1 and PIK3R1 are the core target for the treatment of CIN. The results of molecular docking show that the main active components of *A. manihot* L. have good binding ability to the corresponding core targets. The vitro experiments confirmed that total flavonoids of *A. manihot* L. can significantly reduce iopromide-induced apoptosis of HK2 cells, which may be related to the up-regulation of AKT phosphorylation. In summary, this study preliminarily revealed the multi-component, multi-target, and multi-pathway synergistic effect of *A. manihot* L. on CIN, which provided a theoretical reference and basis for the study of the pharmacological mechanism of *A. manihot* L. in the treatment of CIN.

## Author Contributions

XL conceived and designed the experiments. ZX, YW, and RN collected and analyzed the data. LQ carried out the molecular docking analysis. ZX and YY performed the experiments. ZX and CL wrote the manuscript. XL proofread the manuscript. All authors contributed to the article and approved the submitted version.

## Funding

The authors greatly appreciate the financial support from the National Natural Science Foundation of China (No. 81973762) and Xia Guicheng Innovation and Development Fund of Jiangsu Provincial Hospital of Traditional Chinese Medicine (No. Y19019).

## Conflict of Interest

The authors declare that the research was conducted in the absence of any commercial or financial relationships that could be construed as a potential conflict of interest.

## Publisher’s Note

All claims expressed in this article are solely those of the authors and do not necessarily represent those of their affiliated organizations, or those of the publisher, the editors and the reviewers. Any product that may be evaluated in this article, or claim that may be made by its manufacturer, is not guaranteed or endorsed by the publisher.
