# Supplementary material for: Mechanism of Abelmoschus manihot L. in the Treatment of Contrast-Induced Nephropathy on the Basis of Network Pharmacology Analysis
Source: Front Nephrol. 2022 Apr 22;2:834513. doi: 10.3389/fneph.2022.834513 (PMC10479589; doi:10.3389/fneph.2022.834513)

Supplementary Materials for
"Mechanism of Abelmoschus manihot L. in the Treatment of Contrast-induced Nephropathy on the Basis of Network Pharmacology Analysis"

Zhongchi Xu[1,†], Lichao Qian[2,†], Ruge Niu[1], Yibei Wang[1], Ying Yang[1], Chunling Liu[1], Xin Lin[1*]

1. *Jiangsu Provincial Hospital of Chinese Medicine, Affiliated Hospital of Nanjing University of Chinese Medicine, Nanjing, Jiangsu 210029, China*
2. *Nanjing Hospital of Chinese Medicine, Affiliated Hospital of Nanjing University of Chinese Medicine, Nanjing, Jiangsu 210001, China*

†Both authors contributed equally to this paper.

*Corresponding author: Xin Lin. *E-mail address*: linxin@njucm.edu.cn

***Supplementary Fig.1* |** full gel image for the phosphorylation of AKT

***Supplementary Fig.2* |** full gel image for apoptosis related proteins

***Supplementary Fig.3* |** full image for TUNEL staining assay

Supplementary Figure1 : full gel image for Figure 9B

Fig 9B

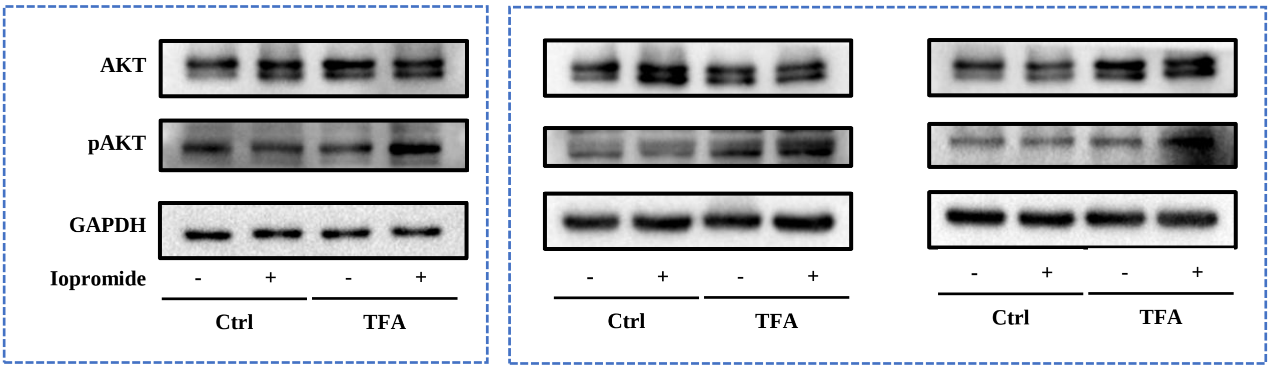

Supplementary Figure2 : full gel image for Figure 9C

Fig 9C

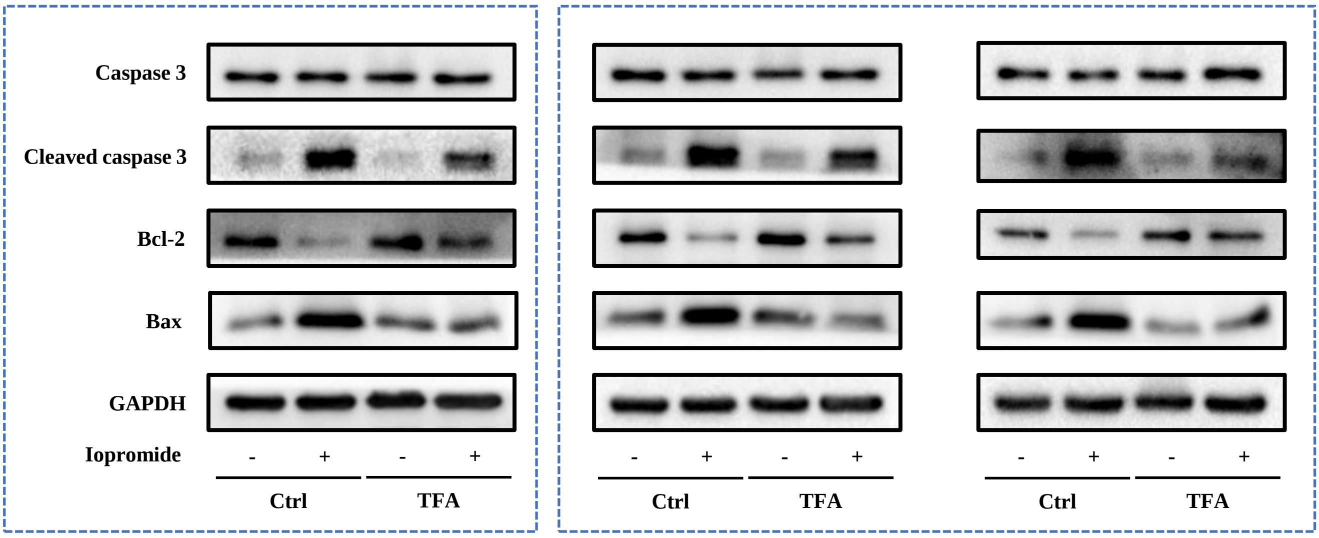

Supplementary Figure3 : full image for Figure 9D

Fig 9D

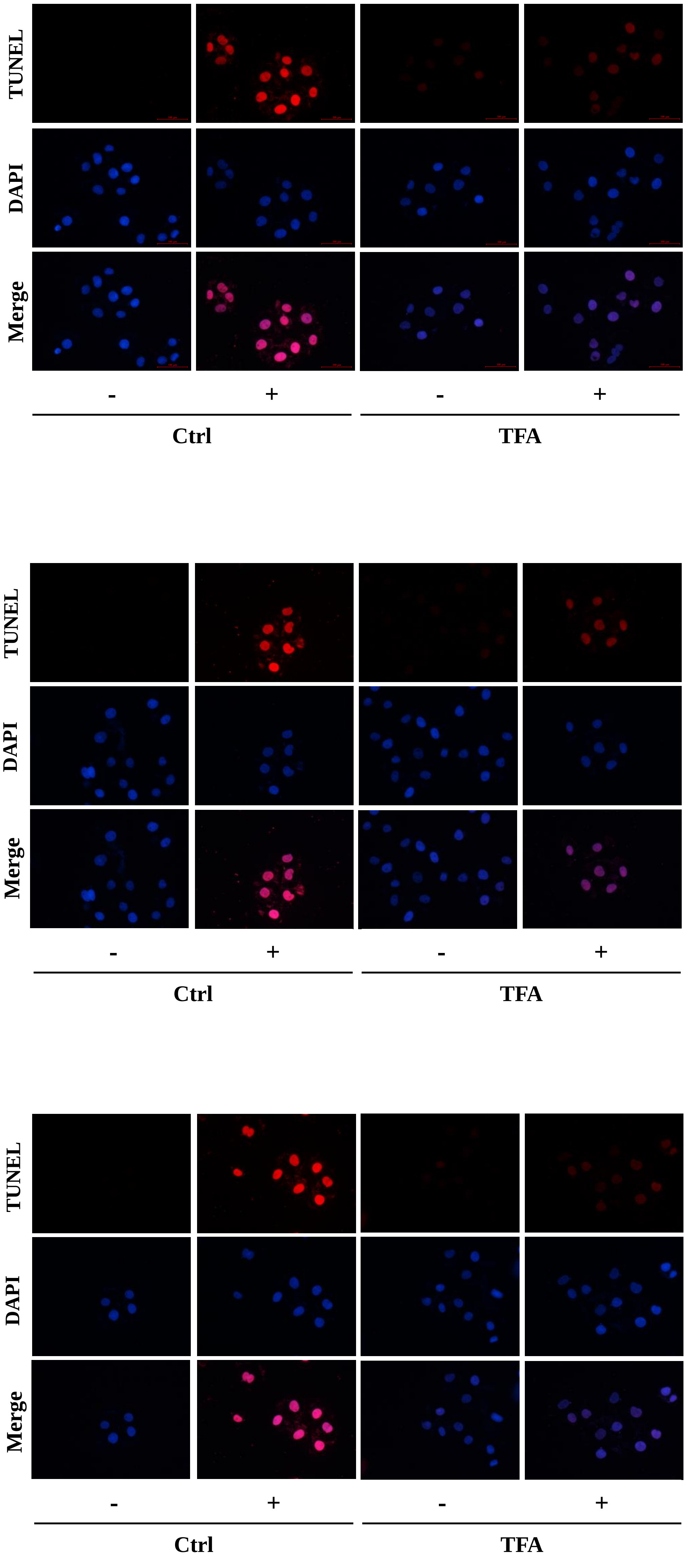

Supplement: Supplementary file 1 [file DataSheet_1.pdf]
